# Genome‐Wide Screening and Functional Verification of the Tobacco SGR Family Identify Transcription‐Factor‐Like NtSGRL as a Negative Regulator of Tomato Yellow Leaf Curl Virus Resistance

**DOI:** 10.1111/mpp.70356

**Published:** 2026-09-29

**Authors:** Ming Gu, Kexin Cao, Jiamin Yu, Dongyang Liu, Bin Zhao, Siyu Sun, Zhenhong Zhu, Mengnan An, Zihao Xia, Zhiping Wang, Yuanhua Wu

**Affiliations:** ^1^ Liaoning Key Laboratory of Plant Pathology, College of Plant Protection Shenyang Agricultural University Shenyang China; ^2^ Key Laboratory of Major Agricultural Invasion Biological Monitoring and Control of Shenyang Shenyang Liaoning China; ^3^ Tobacco Science Research Institute China National Tobacco Corporation Sichuan Branch Chengdu Sichuan China; ^4^ Liangshan Prefecture Branch China National Tobacco Corporation Sichuan Branch Chengdu Sichuan China

**Keywords:** broad‐spectrum antiviral activity, dual‐function protein, negative regulation, STAY‐GREEN (SGR), tomato yellow leaf curl virus (TYLCV)

## Abstract

Viral infection often induces chlorosis, a symptom closely associated with chlorophyll (Chl) degradation. STAY‐GREEN (SGR) proteins are key regulators of Chl breakdown, but their roles in plant virus resistance remain poorly understood. In this study, we identified six *NtSGR* family genes in *Nicotiana tabacum* and functionally characterized *NtSGRL1* during tomato yellow leaf curl virus (TYLCV) infection. TYLCV drastically upregulates *NtSGRL1* (149‐fold at 4 days post‐inoculation [dpi] vs. control), suggesting its involvement in the tobacco–TYLCV interaction. Subcellular localization revealed that NtSGRL1 is targeted to both the nucleus and chloroplast, and yeast assays confirmed its transcriptional activation activity, indicating potential dual functions. TYLCV accumulation was reduced by 1995‐fold in *Ntsgrl1* mutants relative to wild‐type (WT) plants at 10 dpi. Further assays with tomato spotted wilt virus (TSWV), potato virus Y (PVY) and tobacco mosaic virus (TMV) showed that viral accumulation in *Ntsgrl1* mutants was reduced by 62%–99%, suggesting that NtSGRL1 may affect resistance to additional tested viruses. Transcriptomics revealed 1470 differentially expressed genes in TYLCV‐infected *Ntsgrl1* plants enriched in MAPK signalling and phenylpropanoid biosynthesis pathways. Virus‐induced gene silencing (VIGS)‐based validation further supported the involvement of these pathways in TYLCV resistance. Overall, this study identifies *NtSGRL1* as a critical regulator of antiviral resistance in tobacco and reveals its dual functional roles as both a Chl‐related enzyme and a transcriptional regulator. These findings provide new insights into plant antiviral mechanisms and potential genetic resources for breeding virus‐resistant crops.

## Graphical abstract

A chloroplast‐associated STAY‐GREEN protein, NtSGRL1, acts as a negative regulator of antiviral immunity in tobacco. Disruption of NtSGRL1 enhances resistance to diverse plant viruses and reveals a previously unrecognized link between chloroplast regulation and immune signalling.

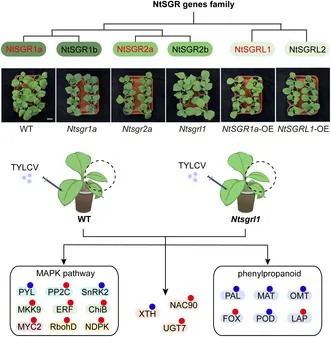

## 1 Introduction

Tomato yellow leaf curl virus (TYLCV) is a highly destructive begomovirus causing severe yield losses in tomatoes worldwide (Cao et al. 2024). Beyond tomatoes, TYLCV infects a range of solanaceous crops and weeds that can act as alternative hosts, serving as reservoirs for viral persistence and dissemination (Kil, Byun, et al. 2014; Kil, Park, et al. 2014; Kil et al. 2015; Smith et al. 2015). TYLCV is transmitted by the whitefly *Bemisia tabaci* , which facilitates rapid virus spread across crops and regions, posing significant challenges for disease control (Xie et al. 2024). Previous studies have shown that flue‐cured tobacco (*Nicotiana tabacum*) can serve as a reservoir host for TYLCV, from which the *B. tabaci* can readily acquire the virus, thereby potentially contributing substantially to the spread of TYLCV epidemics in China (Li et al. 2017). Given the dual role of tobacco as both a potential natural host and a model system, it is of critical importance to elucidate the mechanisms of tobacco resistance to TYLCV.

Viral invasion primarily disrupts the ultrastructure of chloroplasts, impairs the function of the photosystem, and accelerates the degradation of Chl and photosystem‐associated proteins, which is closely associated with chlorosis symptoms (Domínguez and Cejudo 2021; Li et al. 2021; Jiang and Zhou 2023). Mechanistically, virus‐induced chlorosis is mediated via at least three interconnected processes: the degradation of Chl‐binding proteins, the suppression of Chl biosynthesis, and the disruption of chloroplast integrity (Zhao et al. 2016). Chl‐related genes and proteins (CPRGs/CPRPs) are thus considered major targets of viral interference. For instance, a 22‐nt siRNA derived from cucumber mosaic virus Y satellite RNA (CMV‐Y‐sat) specifically targets the transcripts of the magnesium protoporphyrin chelatase subunit I (ChlI), a key enzyme in Chl biosynthesis, resulting in its silencing and a characteristic bright yellow mosaic phenotype (Shimura et al. 2011; Smith et al. 2011). Chl catabolism is emerging as a critical regulatory hub linking chloroplast homeostasis and plant immunity. A recent study in maize demonstrated that disruption of the *LETHAL LEAF SPOT1* (*LLS1*), a pheophorbide a oxygenase involved in Chl breakdown, leads to a lesion mimic phenotype accompanied by broad‐spectrum disease resistance. Disruption of this pathway can convert metabolic intermediates into potent pro‐death signals, thereby reshaping immune outputs (Li et al. 2022). In addition, infection by the tobacco mosaic virus (TMV) flavum strain causes the depletion of Photosystem II (PSII) core complexes and oxygen‐evolving complex (OEC) components, such as chloroplast‐encoded PsbA, nuclear‐encoded Chl *a*/*b*‐binding proteins LhcB1/LhcB2, and PsbO, directly impairing Chl function and photosynthesis and representing a key mechanism underlying viral chlorosis (Lehto et al. 2003). At the functional level, these structural and molecular perturbations are accompanied by alterations in Chl fluorescence parameters, specifically a reduction in the maximum quantum efficiency of PSII (Fv/Fm), which indicates compromised photosynthetic performance (Rahoutei et al. 2001; Breen et al. 2022). Collectively, these findings demonstrate that virus‐induced chlorosis is not merely a passive symptom of infection but rather reflects an actively regulated process involving coordinated disruption of chloroplast structure, Chl metabolism, and photosynthetic function.

Concurrently, the virus‐induced accumulation of reactive oxygen species (ROS) triggers oxidative stress, which in turn promotes chloroplast dismantling and the progression of leaf senescence (Xu et al. 2024). Viral infection also disrupts phytohormone homeostasis, especially that of ethylene (ET), abscisic acid (ABA) and jasmonic acid (JA), thereby activating senescence‐associated pathways (Sharma and Prasad 2025; Islam et al. 2023). At the molecular level, senescence‐associated genes (SAGs) and key transcription factors, including members of the NAC and WRKY families, are modulated, reinforcing senescence progression (Espinoza et al. 2007; Dong et al. 2024; Wang et al. 2026). Notably, Chl degradation not only marks leaf senescence but also participates in a positive feedback loop that further drives the process (Sakuraba et al. 2015).

The STAY‐GREEN (SGR) family is a key regulatory module in the plant Chl degradation pathway. Its functions extend beyond senescence‐related Chl catabolism to include interactions with hormone signalling, ROS homeostasis and stress responses (Kusaba et al. 2013; Hayashi et al. 2024; Tanaka and Ito 2025). Intriguingly, several studies have implicated SGR in disease resistance beyond its canonical role in senescence. For instance, the *Arabidopsis* mutant *noc1*, which carries a mutation in the *SGR* gene (also known as Mendel's *I* locus), exhibits attenuated disease symptoms upon infection with the fungal pathogen *Alternaria brassicicola* (Armstead et al. 2007; Mecey et al. 2011). In cucumber, a single base mutation in *CsSGR* has been deployed as a broad‐spectrum disease resistance gene in breeding programmes for over 50 years (Wang et al. 2019). In rice, the mutation of *OsSGR* significantly enhances the resistance to rice sheath blight by downregulating the cytokinin‐degrading *OsCKX* genes, thereby increasing the cytokinin levels (Xie et al. 2024). Collectively, these findings demonstrate that SGR‐mediated Chl catabolism can, depending on the context, modulate plant immunity against fungal and bacterial pathogens, often through the homeostasis of ROS and phytohormones. However, it remains entirely unknown whether SGR family members play a role in defence against viral infections.

Because the SGR proteins are central regulators of Chl breakdown, the Chl retention caused by *SGR* mutation may, to a certain extent, delay the viral infection process or enhance the resistance of host plants to viruses. Based on this hypothesis, we conducted a genome‐wide identification of the *SGR* gene family members in *N. tabacum* . The fundamental biological functions of different members of the *NtSGR* gene family and their potential roles during virus infection were systematically investigated through transgenic functional verification. Based on the analysis of gene expression levels upon TYLCV infection, we found that *NtSGRL1* exhibited the most robust response to TYLCV. By integrating molecular, genetic, and biochemical approaches, we demonstrate that *NtSGRL1* negatively regulates broad‐spectrum resistance to multiple viruses in tobacco. RNA sequencing (RNA‐seq) analyses revealed transcriptional enrichment of genes annotated to MAPK signalling‐ and phenylpropanoid biosynthesis‐related categories, and virus‐induced gene silencing (VIGS) assays supported the involvement of selected candidate genes from these categories in tobacco resistance to TYLCV infection. Importantly, the knockout of *NtSGRL1* enhanced broad‐spectrum antiviral resistance and, at the same time, avoided the stay‐green phenotype without tobacco yield penalty, showing a great potential for practical application.

## 2 Results

### 2.1 Phylogenetic and Molecular Characteristics of the Tobacco NtSGR Gene Family

We identified a total of six potential SGR proteins in tobacco through a BlastP search of the query AtSGR protein family sequences against the *N. tabacum* K326 genome (Sierro et al. 2014) (Table S1). Phylogenetic analysis, which was performed together with representative SGR proteins from *Arabidopsis*, rice, soybean, maize, pepper, and tomato, resolved the NtSGR proteins into two well‐supported clades corresponding to the canonical SGR and SGRL lineages (Figure 1A, Table S2). The six candidate tobacco SGR proteins were named according to the homologous proteins in *Arabidopsis thaliana* . As *N. tabacum* is an allotetraploid (2n = 4x = 48) that originated through allopolyploidization, which involves interspecific hybridization and subsequent genome doubling between two diploid progenitors, *N. sylvestris* (2n = 2x = 24) and *N. tomentosiformis* (2n = 2x = 24), it possesses an SSTT genomic constitution. The majority of conserved functional genes retain two homologous copies in the S and T subgenomes. Accordingly, based on sequence homology, we designated these six NtSGR proteins as NtSGR1a/b, NtSGR2a/b and NtSGRL1/2, respectively.

**FIGURE 1:**
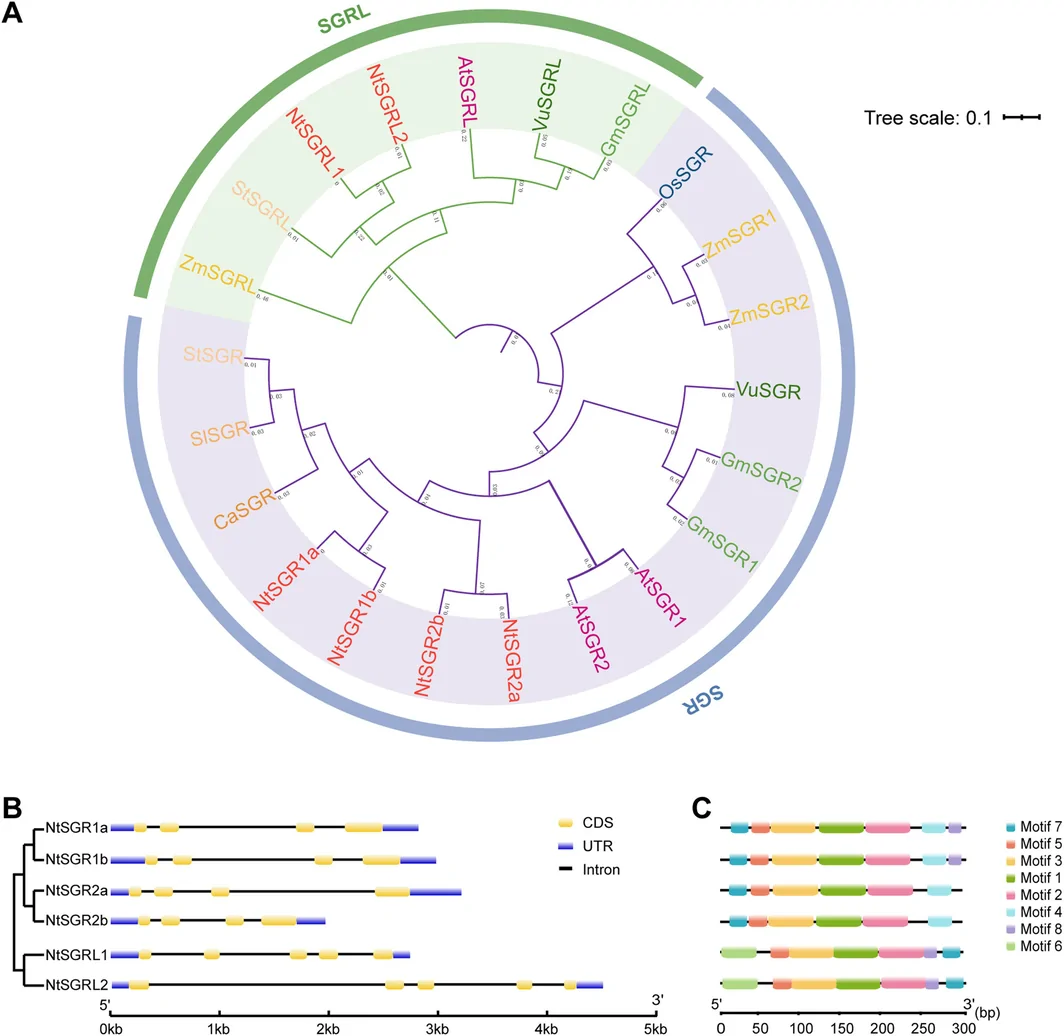
Phylogenetic relationships and structures features of the *Nicotiana tabacum* (tobacco) NtSGR family. (A) Phylogenetic analysis of NtSGR proteins and representative STAY‐GREEN family members from *Arabidopsis thaliana* (At), *Oryza sativa* (Os), *Glycine max* (Gm), *Zea mays* (Zm), *Capsicum annuum* (Ca) and *Solanum lycopersicum* (Sl). NtSGR proteins are highlighted in red. The phylogenetic tree was generated using the neighbour‐joining method with a bootstrap value of 1000. Branches supported with bootstrap values above 70 have increased thickness. Scale bar represents branch length. (B) Gene structure analysis of *NtSGR* family genes. Exons are represented by yellow boxes, introns by black lines and untranslated regions (UTRs) by blue boxes. Gene lengths are shown relative to the scale at the bottom. (C) Distribution of conserved motifs. Motifs were identified using MEME and are represented by coloured boxes.

Multiple‐sequence alignment showed that all NtSGR proteins preserve the characteristic STAY‐GREEN architecture, which encompasses an N‐terminal chloroplast transit peptide and a highly conserved central STAY‐GREEN domain (Figure S1). In contrast, the C‐terminal region, specifically around the cysteine‐rich motif (CRM), exhibited pronounced sequence variability among family members. Consistently, the predicted tertiary structures revealed a conserved overall fold dominated by the central domain, with structural variation largely confined to the flexible terminal regions (Figure S2). Notably, NtSGRL1 and NtSGRL2 displayed more divergent C‐terminal conformations, which supported their separation from canonical SGR proteins. Gene structure analysis further supported the evolutionary relationships within the NtSGR family (Figure 1B). All six genes shared a conserved exon‐intron organization in terms of the number, although gene lengths varied substantially. *NtSGR1a/1b* and *NtSGR2a/2b* exhibited nearly identical structures, which are consistent with their close phylogenetic relationships. In contrast, *NtSGRL1* and *NtSGRL2* show significant differences in genomic structure. Specifically, *NtSGRL2* possesses longer intron regions than *NtSGRL1*, resulting in a less compact overall structure of *NtSGRL2* compared with that of *NtSGRL1*.

Conserved motif analysis identified eight motifs shared among NtSGR proteins (Figure 1C, Table S3). InterProScan annotation (https://www.ebi.ac.uk/interpro/search/sequence/) showed that several core motifs mapped to the predicted STAY‐GREEN domain (Pfam: PF07109), whereas motifs located mainly at the N‐ and C‐terminal regions showed no significant similarity to known domains. Members within the same subfamily exhibited nearly identical motif composition and order. NtSGR1a/1b and NtSGR2a/2b displayed indistinguishable motif patterns, while NtSGRL1 and NtSGRL2 retained the core motifs but either lacked or rearranged several motifs present in the SGR proteins, especially at the N‐ and C‐terminal regions. These findings suggest that the SGR and SGRL subfamilies share a conserved functional core but have undergone subfamily‐specific structural diversification.

### 2.2 The NtSGR Gene Family Exhibits Distinct Functional Divergence in Regulating Tobacco Leaf Senescence and Modulating Chl Degradation Efficiency

In *Arabidopsis*, SGR1, SGR2 and SGRL have been functionally characterized, showing both conserved and divergent roles in Chl catabolism (Sakuraba et al. 2014; Sakuraba et al. 2015). However, whether these functional relationships are conserved in other species, such as in tobacco, remains largely unclear. To address this question, we examined the expression patterns of *NtSGRL1*, *NtSGR1a* and *NtSGR2a* during plant development and senescence. Tobacco seedlings were transferred from Murashige and Skoog (MS) medium to soil, and sampling was initiated 4 weeks after transplantation, which was designated as 0 week; subsequent samples were collected at weekly intervals thereafter. *NtSGRL1* displayed an expression pattern distinct from those of *NtSGR1a* and *NtSGR2a*, with its transcript level increasing from 0 week, peaking at 1 week, and subsequently declining (Figure 2A). By contrast, *NtSGR1a* and *NtSGR2a* were rapidly upregulated, with *NtSGR1a* showing the most dramatic increase. These distinct expression patterns suggest functional divergence among *NtSGR* family members during leaf development and senescence, with *NtSGRL1* differing from the canonical senescence‐associated expression patterns of *NtSGR1a* and *NtSGR2a*.

**FIGURE 2:**
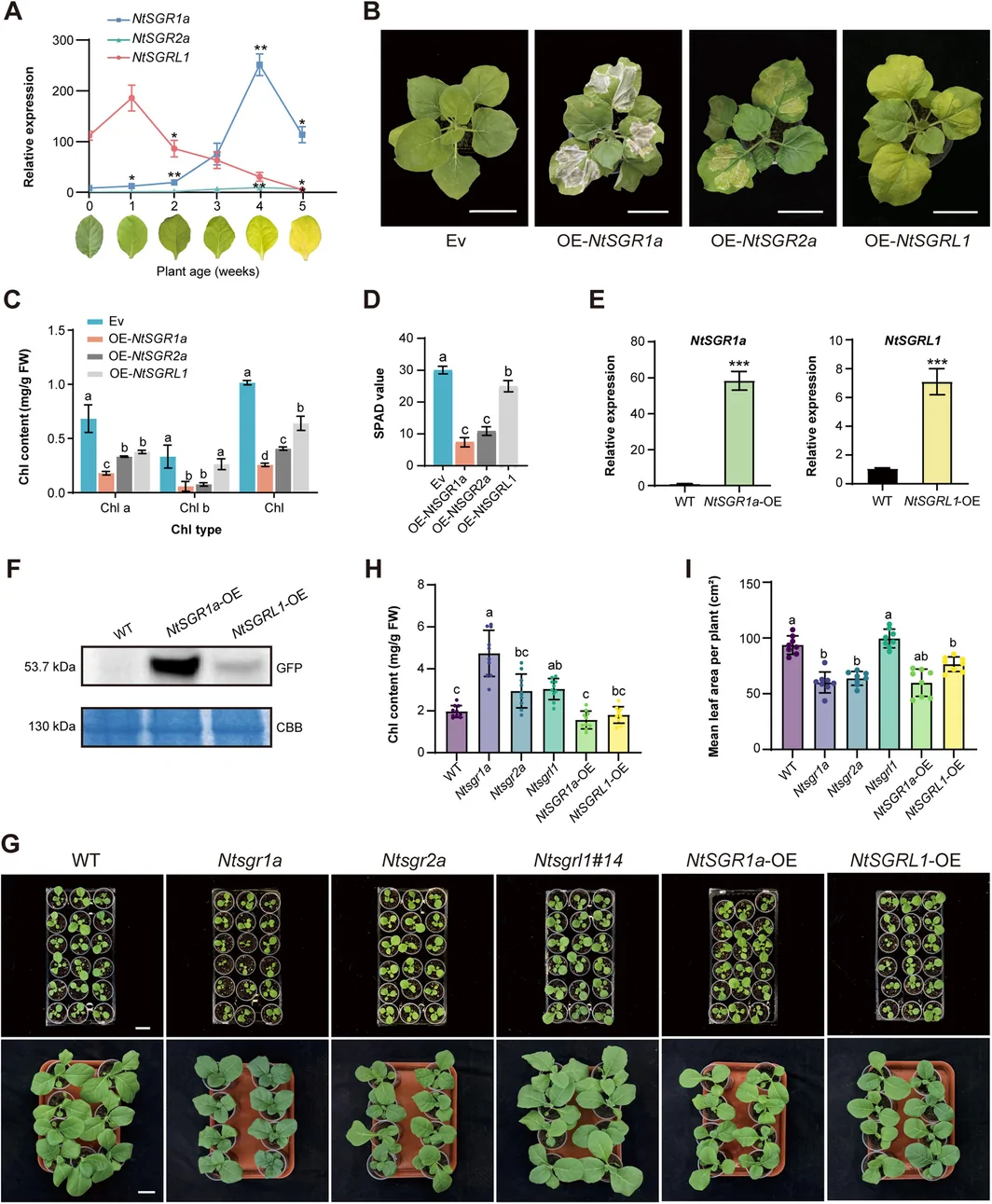
Expression dynamics and functional characterization of *NtSGR* gene family members in *Nicotiana tabacum* (tobacco). (A) Relative expression levels of *NtSGR1a*, *NtSGR2a*, and *NtSGRL1* during natural leaf senescence at indicated time points (0–5 weeks). Data represent means ± SD (*n* = 3). Asterisks indicate significant differences compared with previous time point (*p < 0.05, **p < 0.01). (B) Phenotypic changes of tobacco leaves transiently expressing empty vector (EV), *NtSGR1a*, *NtSGR2a* or *NtSGRL1*. Representative images were taken at 60 h after infiltration. (C, D) Quantification of chlorophyll content and SPAD values in leaves expressing EV or *NtSGR* homologues. Different letters indicate statistically significant differences (*p* < 0.05, one‐way ANOVA). Data represent means ± SD (*n* = 3). (E) Expression analysis of *NtSGR1a* and *NtSGRL1* in wild‐type (WT) and the corresponding stable overexpression (OE) lines by reverse transcription‐quantitative PCR. Significant difference, ***p < 0.001. (F) Detection of the NtSGRL1‐GFP fusion protein in *NtSGR1a* and *NtSGRL1*‐OE lines by western blotting using an anti‐GFP antibody and Coomassie Brilliant Blue staining (CBB) as a loading control. (G) Phenotypic comparison of WT, *Ntsgr1a*, *Ntsgr2a* and *Ntsgrl1* mutants, as well as stable *NtSGR1a*‐OE and *NtSGRL1*‐OE transgenic lines under greenhouse conditions. (H) Total chlorophyll content of WT, mutant, and OE lines. (I) Mean leaf area per plant of WT, mutant and OE lines. Numbers indicate relative protein abundance between two OE lines. Data represent means ± SD (*n* = 8). Different lowercase letters above the bars indicate statistically significant differences among genotypes as determined by one‐way ANOVA followed by Tukey's multiple comparison test (*p* < 0.05).

Transient overexpression assays further demonstrated that *NtSGRL1*, together with *NtSGR1a* and *NtSGR2a*, promoted leaf yellowing (Figure 2B, Figure S3). However, the degree of chlorosis differed among the three genes, with *NtSGR1a* showing the strongest effect, followed by *NtSGR2a*, and *NtSGRL1* exhibiting a weaker phenotype. Consistently, both SPAD values and Chl contents (Chl *a*, Chl *b*, and total Chl) were significantly reduced in all overexpression (OE) lines (Figure 2C,D). These results are consistent with previous findings in *Arabidopsis*, showing that overexpression of *SGRL* accelerates leaf yellowing and Chl degradation, which indicates that the Chl‐degrading function of *SGRL* is evolutionarily conserved across plant lineages. Additionally, hormone treatment experiments showed that *NtSGR1a* was highly responsive to both abscisic acid (ABA) and methyl jasmonate (MeJA), while *NtSGRL1* exhibited limited sensitivity to these two signalling pathways (Figure S4). This suggested that *SGR1* is tightly linked to senescence‐ and hormone‐regulated pathways, while *SGRL* displays a more context‐dependent expression pattern.

To further validate their biological functions, we generated stable genetic materials, including CRISPR/Cas9‐mediated knockout mutants and stable OE lines. Three homozygous mutant lines carrying frameshift mutations that introduced premature termination codons were selected for *Ntsgr1a*, *Ntsgr2a* and *Ntsgrl1* for subsequent phenotypic analyses (Figure S5). In parallel, *NtSGR1a*‐OE and *NtSGRL1*‐OE transgenic lines were generated, and elevated transcript and protein accumulation of the corresponding genes was confirmed by reverse transcription‐quantitative PCR (RT‐qPCR) and western blotting, respectively (Figure 2E,F). Compared with WT, *Ntsgr1a* mutants displayed greener leaves and growth inhibition, indicating that *NtSGR1a* plays dominant roles in Chl degradation and potentially exerts strong pleiotropic effects on plant development; the *Ntsgrl1#14* mutants exhibited slightly improved biomass accumulation and leaf expansion (Figure 2G). Conversely, *NtSGR1a*‐OE plants exhibited severe chlorotic phenotypes and reduced leaf growth, while *NtSGRL1*‐OE plants showed only moderate chlorosis. These phenotypic differences were further supported by Chl quantification and growth performance analysis, confirming that alterations in *NtSGR* expression significantly affected leaf development and biomass accumulation. Collectively, these findings demonstrate that members of the *NtSGR* gene family exhibit both functional conservation and clear divergence in regulating Chl degradation and leaf senescence in tobacco. Among them, *NtSGR1a* functions as a dominant regulator of Chl catabolism, whereas *NtSGRL1* displays a distinct temporal expression pattern characterized by preferential induction at early developmental stages and reduced responsiveness to senescence‐ and hormone‐associated signals.

### 2.3 NtSGRL1 Exhibits Significant Volatility Before and After TYLCV Infection

To explore the functional properties of NtSGR proteins, we first examined their subcellular localization. Subcellular localization analysis showed NtSGRL1, NtSGR1a, and NtSGR2a signals were predominantly co‐localized with chloroplast autofluorescence, consistent with the canonical role of SGR proteins in Chl catabolism within chloroplasts (Figure 3A). In addition, co‐localization with an RFP nuclear marker demonstrated that GFP signals from NtSGRL1 and NtSGR2a overlapped with nuclear marker signals, whereas NtSGR1a showed no obvious overlap under our experimental conditions (Figure 3B). The nuclear localization of NtSGRL1 and NtSGR2a suggests they may possess additional regulatory roles beyond chloroplast‐localized metabolic functions (Figure 3B). Given that SGR proteins function as terminal regulators of Chl catabolism, these findings raise the possibility that NtSGRL1 and NtSGR2a may also participate in broader stress‐responsive signalling processes, which prompted further investigation of their potential roles in host–virus interactions.

**FIGURE 3:**
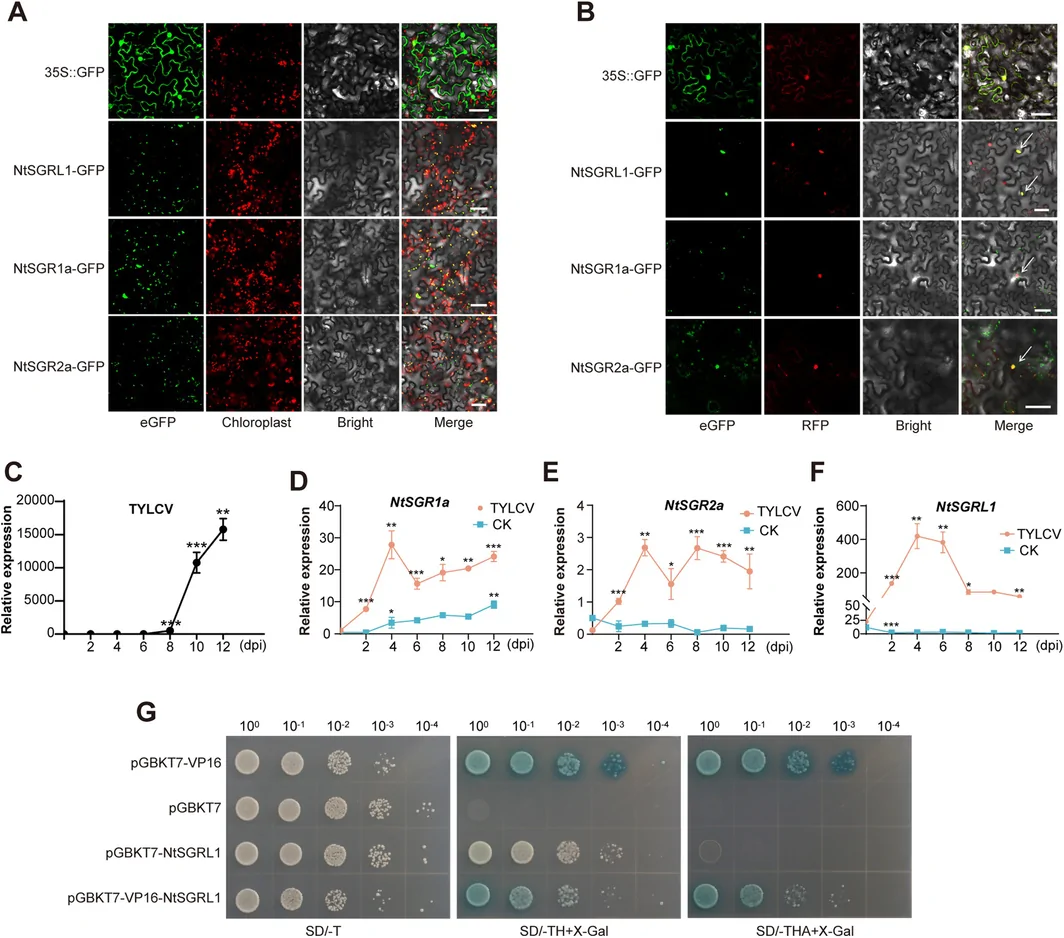
Subcellular localization, TYLCV‐responsive expression dynamics, and transcriptional activity of NtSGR proteins. (A, B) Subcellular localization of NtSGR1a, NtSGR2a and NtSGRL1 fused to GFP in *Nicotiana tabacum* (tobacco) epidermal cells. Chloroplasts were visualized by autofluorescence, and nuclei were labelled with RFP. GFP signals were observed by confocal microscopy. Scale bar = 50 μm. (C) Time‐course analysis of TYLCV accumulation in systemic leaves following infection. (D–F) Expression profiles of *NtSGR1a*, *NtSGR2a*, and *NtSGRL1* in response to TYLCV infection at different time points. CK indicates mock‐inoculation treatment. (G) Yeast transcription activation assay analysing the transcriptional activation activity of NtSGRL1. Data represent means ± SD (*n* = 3). Asterisks indicate significant differences compared with the previous day (*p < 0.05, **p < 0.01, ***p < 0.001).

To this end, we evaluated the transcriptional responsiveness of *NtSGR* genes during virus infection. TYLCV was selected as a representative DNA virus because it is a globally prevalent pathogen that causes severe chlorosis and growth arrest in solanaceous crops, phenotypes that are closely associated with Chl metabolism and chloroplast dysfunction. RT‐qPCR assays revealed that all three genes responded to viral infection but exhibited distinct expression dynamics. Viral accumulation increased sharply after 8 days post‐inoculation (dpi) and reached a peak at 10–12 dpi (Figure 3C). Among the tested genes, *NtSGR1a* showed a rapid transient induction at early infection stages (2–4 dpi) followed by a decline, whereas *NtSGR2a* exhibited moderate and fluctuating upregulation throughout infection (Figure 3D,E). Notably, *NtSGRL1* displayed the strongest and most sustained induction, with a rapid increase beginning at 2 dpi and remaining at significantly higher levels than the mock‐inoculation control (CK) across all later time points (Figure 3F). The expression amplitude of *NtSGRL1* was markedly higher than that of *NtSGR1a* and *NtSGR2a*. These results suggest that NtSGRL1 may play a more prominent role in early host–virus interaction processes compared with other canonical NtSGR family members.

To determine whether *NtSGRL1* has regulatory activity beyond its putative enzymatic role, its transcriptional activation activity was examined in yeast. *NtSGRL1* was fused to the GAL4 DNA‐binding domain in pGBKT7 and transformed into a reporter yeast strain. The pGBKT7‐NtSGRL1 transformants grew on selective medium and produced a positive colorimetric signal, whereas the empty vector did not. VP16, a strong transcriptional activation domain, was used as a positive control to verify reporter activation. These results indicate that *NtSGRL1* has transcriptional activation activity in yeast, supporting its potential regulatory function (Figure 3G).

### 2.4 NtSGRL Negatively Regulates the Resistance of *N. tabacum* Against TYLCV Without Biomass Loss

To elucidate the biological function of *NtSGRL1*, we selected three knockout lines and an OE line (Figure S6A,B). The three homozygous gene‐edited lines including a 3‐bp deletion (*Ntsgrl1*#1), a 1‐bp insertion (*Ntsgrl1*#8) and a 5‐bp deletion (*Ntsgrl1*#14) at the target site. Sequence analysis revealed that *Ntsgrl1*#1 resulted in amino acid substitutions, while *Ntsgrl1*#8 and *Ntsgrl1*#14 induced frameshift mutations resulting in a truncated protein (Figure S6A). Phenotypic differences were already quite evident during the seedling stage. Compared with the wild type (WT), *Ntsgrl1* mutants exhibited enhanced seedling vigour, characterized by increased leaf expansion and overall biomass accumulation, whereas OE plants displayed reduced growth (Figure S6C). These results suggest that loss of *NtSGRL1* function promotes early leaf expansion during the seedling stage, while its overexpression suppresses early development.

To study the function of *NtSGRL1* in antiviral defence, we inoculated WT, *NtSGRL1‐*OE, and *Ntsgrl1* mutants with TYLCV and scored their disease severity at 10 and 20 dpi. Prior to inoculation, the overall growth vigour of all genotypes was essentially uniform (Figure 4A). At 10 dpi, OE plants exhibited pronounced leaf curling and growth retardation, whereas WT plants showed intermediate symptoms and *Ntsgrl1* mutants displayed only mild phenotypic alterations. These differences became more pronounced by 20 dpi, with OE plants showing severe developmental defects and *Ntsgrl1* lines remaining relatively less affected (Figure 4A,B). Consistent with symptom severity, TYLCV DNA and coat protein (CP) accumulation were significantly higher in OE plants compared to WT, while *Ntsgrl1* mutants accumulated markedly lower viral transcripts at both 10 and 20 dpi (Figure 4C,D). These observations indicate that the loss of *NtSGRL1* function mitigates the symptoms caused by TYLCV, whereas its overexpression exacerbates the disease phenotype.

**FIGURE 4:**
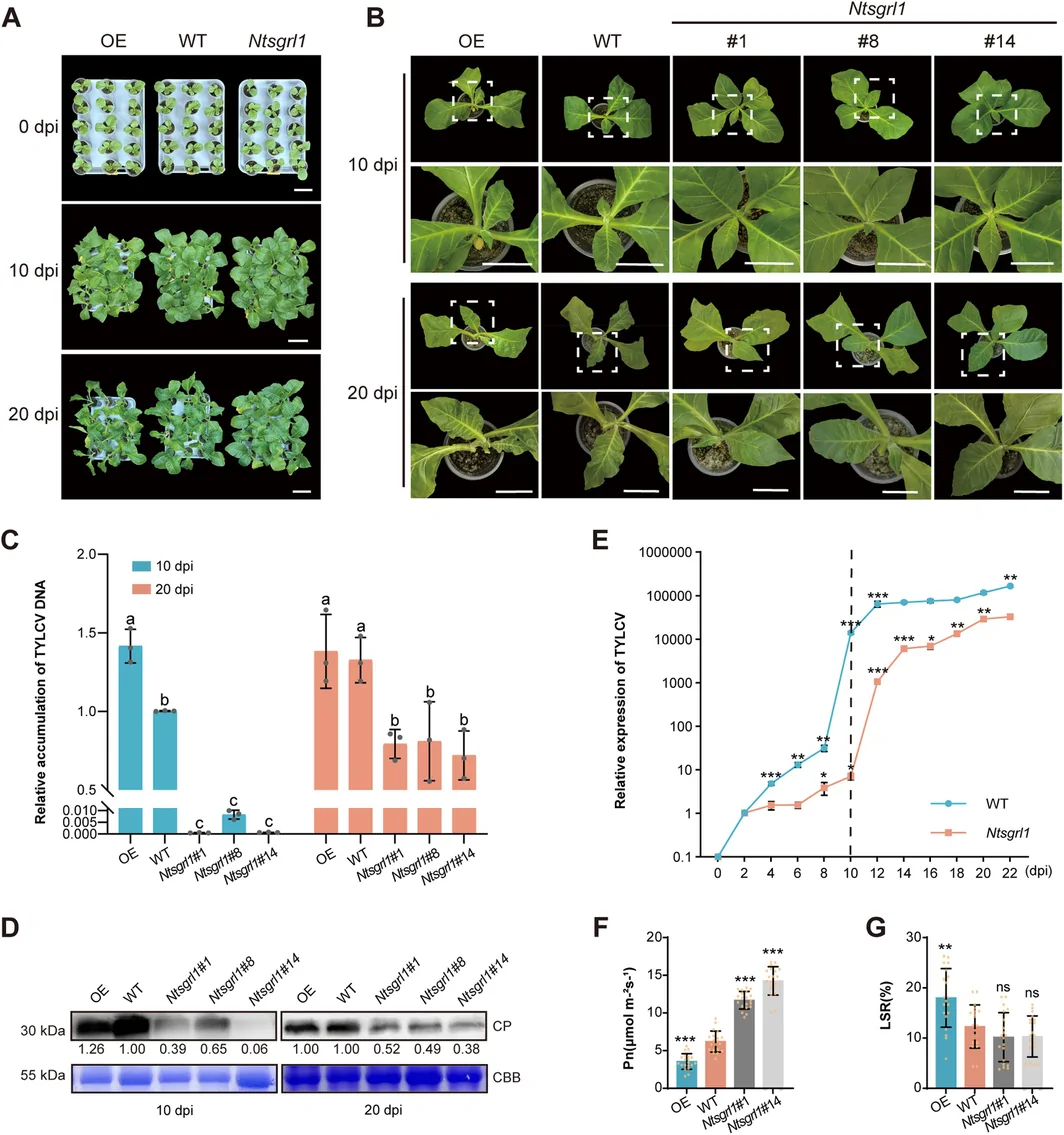
Knockout of *NtSGRL1* significantly compromised TYLCV accumulation in non‐inoculated upper leaves. (A) Group phenotypic occurrence of wild type (WT), *Ntsgrl1* mutants, and *NtSGRL1*‐OE (overexpression) lines at 0, 10, and 20 days post‐inoculation (dpi) with TYLCV. Scale bar = 10 cm. (B) Comparison of disease severity based on leaf curling and growth retardation at indicated time points. Scale bar = 5 cm. Enlarged views of upper leaves (highlighted by white dashed boxes) are shown below. (C) Virus accumulation in different genotypes inoculated with TYLCV. Virus accumulation in the non‐inoculated upper leaves at 10 and 20 dpi was assessed using reverse transcription‐quantitative PCR. (D) Accumulation of TYLCV coat protein (CP) in upper leaves of *Nicotiana tabacum* determined at 10 and 20 dpi by western blotting. Numbers indicate relative protein abundance normalized to WT. Coomassie Brilliant Blue (CBB) staining served as loading control. (E) Time‐course analysis of viral accumulation in WT and *Ntsgrl1* mutant plants following TYLCV infection. The *y*‐axis is displayed on a log_10_ scale. Data in (C) and (E) are presented as means ± SD (*n* = 3). Asterisks indicate significant difference between treatments, determined by the two‐tailed *t*‐test (*p < 0.05, **p < 0.01, ***p < 0.001); “ns” denotes no significant difference. (F, G) Statistically significant results from field trials. Net photosynthetic rate (Pn) in OE, WT and *Ntsgrl1*(#1, 14) at growth stage. Leaf senescence rate (LSR) among WT, and *Ntsgrl1* mutant plants. Data in (F), and (G) are presented as means ± SD (*n* = 20). Asterisks indicate statistical difference between treatments, determined by the two‐tailed *t*‐test (**p < 0.01, ***p < 0.001); “ns” denotes no significant difference.

During the initial stages (0–2 dpi), TYLCV DNA levels were extremely low in both WT and *Ntsgrl1* plants. However, from 4 to 8 dpi, viral DNA accumulated to significantly higher levels in WT plants than in *Ntsgrl1* mutants, albeit at relatively low overall amounts. A distinct inflection point was observed at 8 dpi: WT plants entered a rapid exponential growth phase, with viral loads surging by several orders of magnitude, whereas *Ntsgrl1* mutants exhibited only a moderate increase. From 10 dpi onward (up to 22 dpi), viral DNA continued to accumulate robustly in both genotypes; however, the viral load in the mutants remained consistently and significantly lower (by 1–2 orders of magnitude) than that in WT plants (Figure 4E). These results indicate that the loss of *NtSGRL1* delays the onset of rapid viral accumulation and restricts overall viral DNA levels during infection.

Field trials were conducted at the vegetative and flowering stages to evaluate agronomic performance under near‐natural conditions. Plant height, stem girth, internode length, total leaf number, maximum leaf area, fresh weight, dry weight and thousand‐seed weight were measured across the different genotypes (Figures S7 and S8). No significant differences were detected between *Ntsgrl1* mutants and WT plants for these agronomic or reproductive traits. In contrast, *Ntsgrl1* plants maintained significantly higher net photosynthetic rates (Pn) than WT plants, whereas OE lines exhibited reduced Pn (Figure 4F). Likewise, no significant differences in the timing or progression of leaf senescence were observed among the different genotypes (Figure 4G). Notably, the growth‐promoting effect of *NtSGRL1* knockout was primarily restricted to the seedling stage. During vegetative growth and flowering, the differences in plant architecture and biomass between *Ntsgrl1* and WT plants became negligible (Figures S7 and S8). Collectively, *NtSGRL1* is strongly induced by TYLCV and acts as a negative regulator of antiviral immunity. Importantly, *NtSGRL1* functions as a transcriptional activator, suggesting that it may regulate downstream defence‐related transcriptional reprogramming. Therefore, *NtSGRL1* is identified as a negative regulator of antiviral immunity in tobacco, and its loss‐of‐function confers enhanced resistance to TYLCV without substantial penalties on overall plant biomass or developmental progression, highlighting its potential as a candidate target for improving viral resistance in solanaceous crops.

### 2.5 TYLCV Infection‐Induced Global Transcriptomic Changes in WT and Ntsgrl1 Mutant Tobacco Plants

To elucidate the molecular basis by which loss of *NtSGRL1* function confers enhanced resistance to TYLCV, this study conducted a transcriptome analysis on four groups of tobacco materials at 10 dpi: WT, TYLCV‐infected WT (WT_V), *Ntsgrl1*, and TYLCV‐infected mutant (*Ntsgrl1*_V) (Figure 5A). In this study, a total of 510,316,710 clean reads were obtained with a Q30 percentage > 94.87% and a GC content between 43.19% and 43.71% from 12 tobacco cDNA libraries (Table S4). The clean reads were then mapped to the tobacco reference genome using HISAT2 software and resulted in 93.65%–95.01% mapped reads and 90.70%–92.53% unique mapped reads (Table S5). The clean reads data were deposited in the National Center for Biotechnology Information (NCBI) database (BioProject PRJNA1466156). The relative expression levels of individual genes found in each sample were normalized using the FPKM values as the box plots (Figure S9A). Correlation clustering analysis demonstrated high consistency among replicates and distinct differences between groups (Figure S9B), indicating that the sequencing data were reliable and suitable for subsequent analyses.

**FIGURE 5:**
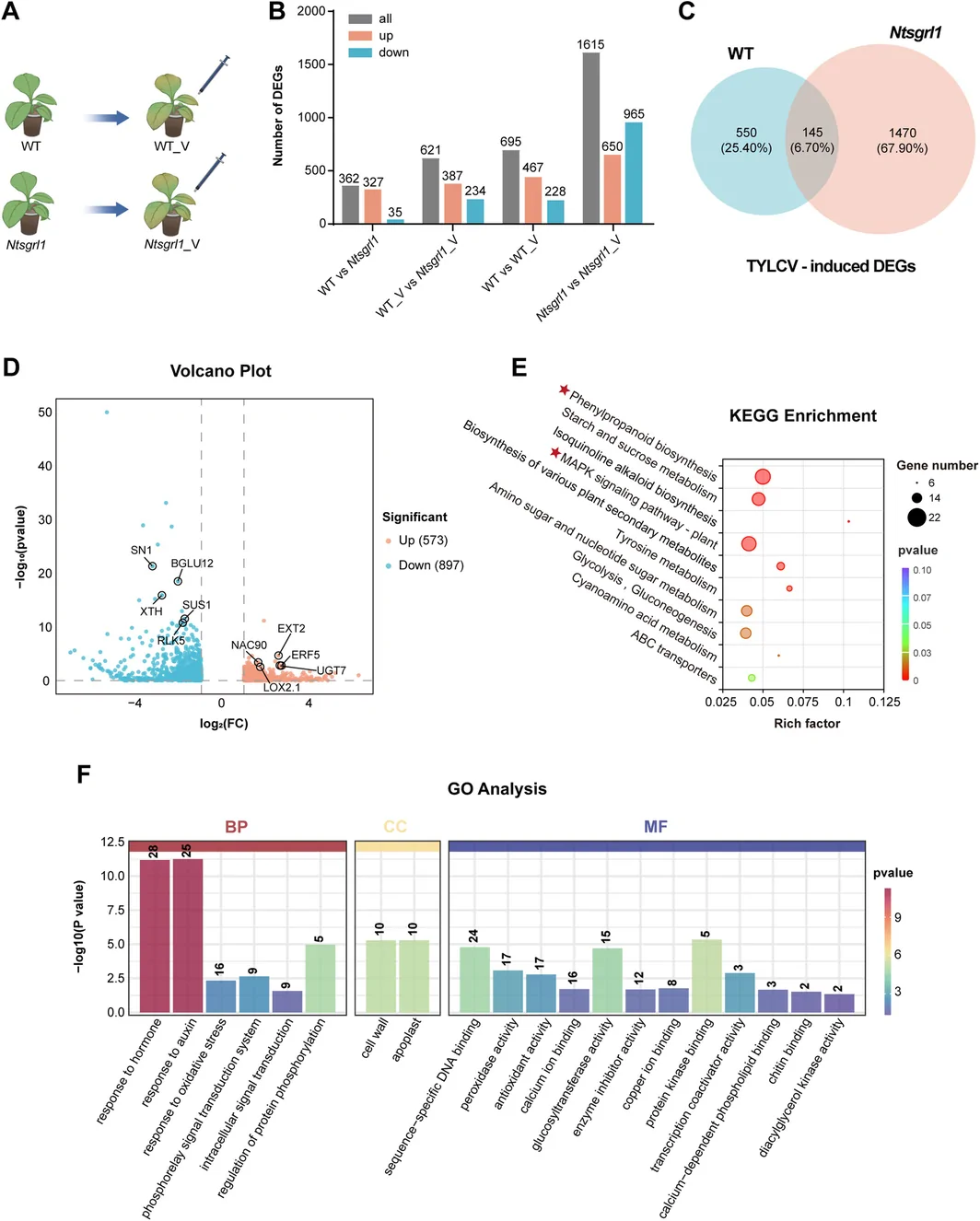
Transcriptome analysis of wild type (WT) and *Ntsgrl1* plants in response to TYLCV infection. (A) Schematic diagram of experimental design showing four sample groups used for RNA‐seq analysis: WT, WT_V (TYLCV‐infected WT), *Ntsgrl1*, and *Ntsgrl1*_V (TYLCV‐infected *Ntsgrl1*). (B) Number of differentially expressed genes (DEGs) identified in each comparison. Grey bars indicate total DEGs, red bars indicate upregulated genes, and blue bars indicate downregulated genes (|log_2_ fold change (FC)| ≥ 1 and *p* < 0.05). (C) Venn diagram showing the number of TYLCV‐induced genes (log_2_ FC| ≥ 1 and *p* < 0.05) in WT and *Ntsgrl1* mutants. (D) Volcano plot of *NtSGRL1*‐dependent TYLCV‐induced genes. Red dots indicate significantly upregulated genes, and blue dots indicate significantly downregulated genes (|log_2_ FC| ≥ 1 and *p* < 0.05). Selected genes with high FC are labelled. (E) KEGG pathways of *NtSGRL1*‐dependent TYLCV‐induced genes. Red stars represent disease resistance‐related KEGG term. (F) Enriched GO categories of *NtSGRL1*‐dependent TYLCV‐induced genes. The number of enriched genes in each GO term is indicated above each bar in the histogram. BP, biological process; CC, cellular component; MF, molecular function.

To investigate the changes of gene expression induced by TYLCV infection in tobacco leaves, we conducted four different pairwise comparisons (i.e., WT vs. *Ntsgrl1*, WT_V vs. *Ntsgrl1*_V, WT vs. WT_V, *Ntsgrl1* vs. *Ntsgrl1*_V). A total of 327 upregulated differentially expressed genes (DEGs) were found in the WT vs. *Ntsgrl1* comparison and were annotated to GO terms under the response to hormone, defence response, cell wall, glucosyltransferase activity, and oxidoreductase activity (Figure 5B, Figure S10A, Table S6). The KEGG pathway analysis result indicated that the DEGs found in the WT vs. *Ntsgrl1* comparison were mainly enriched in carbon metabolism pathways, including the pentose phosphate pathway, fructose and mannose metabolism, and glycolysis/gluconeogenesis, as well as carbon fixation in photosynthetic organisms (Figure S10B, Table S7). In addition, enrichment in amino sugar and nucleotide sugar metabolism, carotenoid biosynthesis, and phenylpropanoid biosynthesis suggests that *NtSGRL1* mutation influences secondary metabolism and cell wall‐related biosynthetic processes.

Following TYLCV infection, a total of 695 transcripts were differentially expressed in WT (fold change ≥ 2, *p* < 0.05), whereas a substantially larger number of transcripts (1615) were differentially expressed in *Ntsgrl1* mutants (Figure 5B). Among these, 1470 transcripts were significantly altered in *Ntsgrl1* mutants compared to WT, including 573 upregulated and 897 downregulated transcripts, which were defined as *NtSGRL1*‐dependent TYLCV‐induced genes (Figure 5C). A volcano plot of *Ntsgrl1*_V vs. *Ntsgrl1* highlighted key transcriptional changes (Figure 5D, Table S8). Several genes related to cell wall modification (*XTH*, *BGLU12*), carbohydrate metabolism (*SUS1*), defence responses (*SN1*), and signal transduction (*RLK5*, *NAC90*) were significantly downregulated, whereas genes involved in stress and hormonal signalling pathways (*EXT2*, *UGT7*, *LOX2.1*, *ERF5*) were upregulated. Intriguingly, a subset of genes classified as downregulated following viral infection exhibited significant upregulation in the WT versus *Ntsgrl1* mutant comparison. This apparent inconsistency may reflect an elevated basal defence‐associated state in the *Ntsgrl1* mutant, characterized by elevated basal levels of defence‐related gene expression prior to infection, thereby potentially underpinning its enhanced antiviral capacity.

To further investigate the response of *Ntsgrl1* mutant leaves to TYLCV infection, we analysed the *NtSGRL1*‐dependent TYLCV‐induced genes using GO and KEGG enrichment analyses. The KEGG pathway enrichment analysis results indicated that the DEGs were mainly enriched in phenylpropanoid biosynthesis, starch and sucrose metabolism, isoquinoline alkaloid biosynthesis, and the MAPK signalling pathway‐plant category (Figure 5E, Table S9). These gene categories are closely linked to cell wall reinforcement, secondary metabolite accumulation, hormone signalling and energy redistribution during antiviral defence. GO classification confirmed that TYLCV‐responsive genes were mainly associated with response to auxin, response to hormone, regulation of protein phosphorylation, response to oxidative stress, cell wall, apoplast, protein kinase activity, glucosyltransferase activity, transcription coactivator activity and calcium‐dependent protein binding (Figure 5F, Table S10). These enriched terms suggest that the enhanced response of *Ntsgrl1* may involve hormone‐mediated signalling, ROS‐associated defence, kinase cascades and cell wall/apoplastic immune responses.

### 2.6 The Enhanced TYLCV Resistance of Ntsgrl1 Is Associated With Transcriptional and Biochemical Changes in Defence‐Related Pathways

To investigate molecular changes associated with the enhanced antiviral phenotype of the *Ntsgrl1* mutant, we examined DEGs related to MAPK signalling and phenylpropanoid biosynthesis (Figure 5E). Genes annotated to ROS‐, ethylene (ET)‐, jasmonic acid (JA)‐, and ABA‐associated processes showed distinct expression patterns between WT and *Ntsgrl1* plants (Figure 6A). Among ROS‐associated genes, *NDPK2* and *RbohD* exhibited higher transcript abundance in the mutant. Consistently, 3,3′‐diaminobenzidine (DAB) staining revealed increased hydrogen peroxide (H_2_O_2_)‐associated accumulation in *Ntsgrl1* plants under both mock and TYLCV‐infected conditions (Figure S11A,B). Western blot analysis further showed increased levels of phosphorylated MAPK proteins in the mutant (Figure S11C), indicating elevated basal MAPK phosphorylation.

**FIGURE 6:**
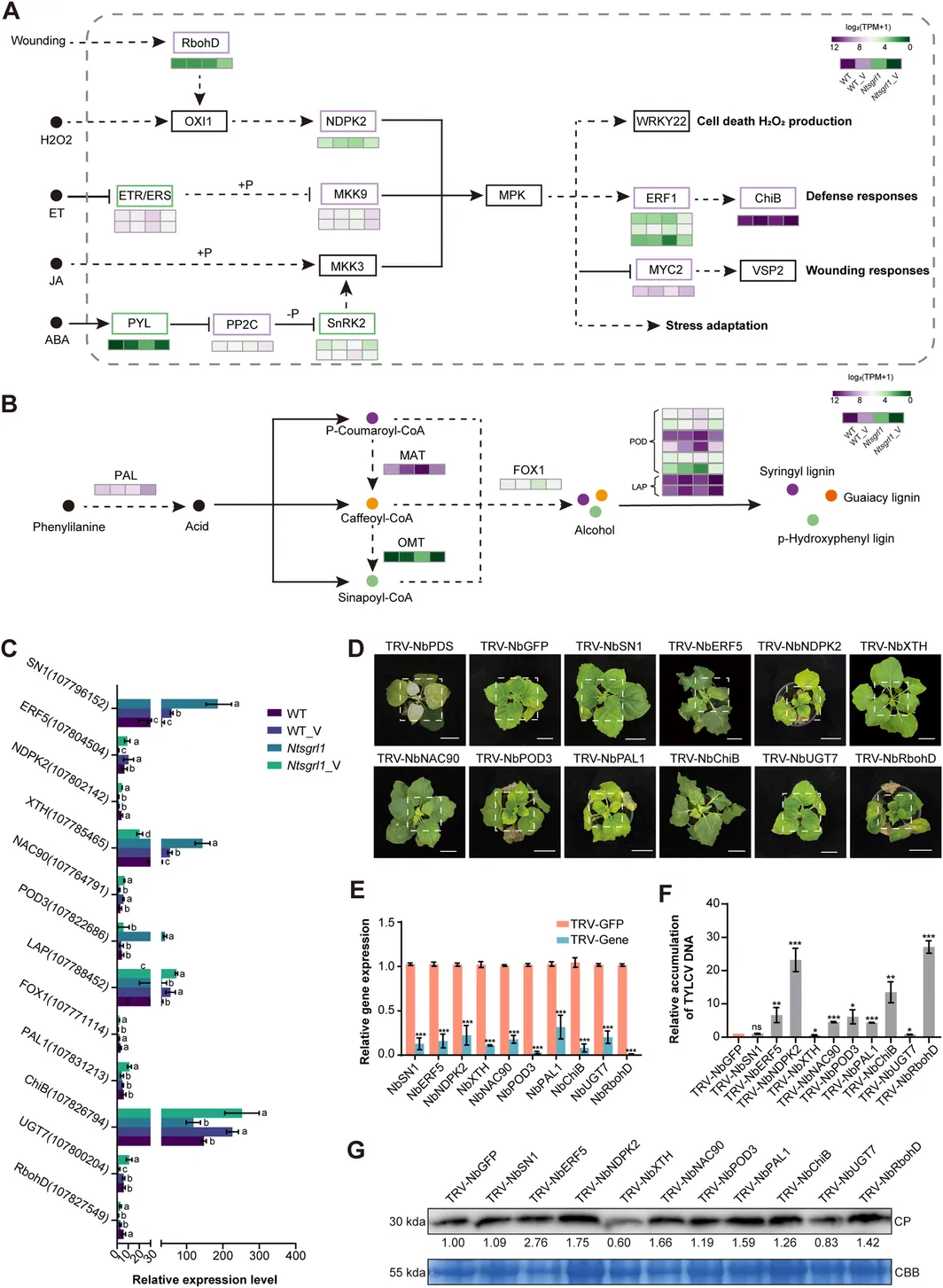
Transcriptional changes in defence‐associated pathways and functional validation of candidate genes in the *Ntsgrl1* mutant. (A) Schematic representation of genes annotated to MAPK signalling pathway with expression patterns of differentially expressed genes (DEGs). Colour scale indicates normalized expression levels (log_2_[TPM + 1]). Genes involved in ethylene signalling, reactive oxygen species (ROS) production, and downstream defence responses are shown. (B) Expression profiles of genes involved in phenylpropanoid biosynthesis. Key enzymes contributing to lignin and secondary metabolite production are highlighted. (C) Relative expression levels of selected candidate genes in wild type (WT), WT_V (TYLCV‐infected), *Ntsgrl1*, and *Ntsgrl1*_V (TYLCV‐infected) plants. Data represent mean ± SD. Different letters indicate statistically significant differences (*p* < 0.05). (D) Phenotypes of *Nicotiana tabacum* (tobacco) plants subjected to virus‐induced gene silencing (VIGS) of candidate genes at 10 days post‐inoculation (dpi). TRV‐GFP was used as a control. (E) Reverse transcription‐quantitative PCR (RT‐qPCR) analysis of virus‐induced gene silencing (VIGS)‐induced target gene silencing efficiencies (*n* = 9). (F, G) Determination of TYLCV accumulation in the leaves from various gene‐silenced tobacco plants or controls at 10 dpi by western blot and RT‐qPCR analyses. CP, coat protein; CBB, Coomassie Brilliant Blue. Results are presented as the mean ± SD. The statistical significance between the treatments was determined using one‐way ANOVA followed by Duncan's multiple comparison test (*p* < 0.05). Asterisks indicate statistical difference between treatments, determined by the two‐tailed *t*‐test (*p < 0.05, **p < 0.01, ***p < 0.001). Scale bar = 5 cm. Abbreviations of genes and compounds are explained in Table S7.

Genes associated with ET‐ and JA‐mediated defence responses, including *ERF1*, *ChiB* and *MYC2*, also showed higher transcript abundance in *Ntsgrl1* plants. Consistent with these transcriptional changes, 1‐aminocyclopropane‐1‐carboxylic acid (ACC) and JA contents were significantly higher in *Ntsgrl1* than in WT plants before and after TYLCV infection (Figure S11D,E). By contrast, the ABA‐associated genes *PYL* and *SnRK2* showed reduced transcript abundance in the mutant, accompanied by lower ABA levels at both basal and post‐infection stages (Figure S11F). Together, these observations indicate that loss of *NtSGRL1* is associated with enhanced ROS accumulation and MAPK phosphorylation, as well as altered ET‐, JA‐, and ABA‐associated responses. Although these data do not establish direct regulatory relationships among these pathways, they support the presence of an altered defence‐associated physiological and transcriptional state in the *Ntsgrl1* mutant.

Genes associated with phenylpropanoid metabolism, including *PAL*, *MAT*, *OMT* and *POD*, were also significantly upregulated in *Ntsgrl1* plants (Figure 6B). These transcriptional changes are consistent with enhanced phenylpropanoid‐ and lignin‐associated metabolism, although direct changes in lignin accumulation were not determined in this study. RT‐qPCR analysis confirmed the expression trends of most genes identified by RNA‐seq (Figure 6C, Table S11). The higher basal expression of several defence‐associated genes further indicates that loss of *NtSGRL1* alters the basal transcriptional state of multiple defence‐related pathways. To functionally assess whether the *NtSGRL1*‐associated transcriptional changes contribute to TYLCV resistance, we selected 10 representative candidate genes from the *NtSGRL1*‐responsive defence network for VIGS analysis in *Nicotiana benthamiana*. These genes were chosen to cover several major defence‐related processes enriched in the transcriptomic analysis, including ROS production and redox signalling (*NbRbohD* and *NbNDPK2*), hormone‐associated defence regulation (*NbERF5* and *NbChiB*), phenylpropanoid metabolism and cell wall‐related responses (*NbPAL1*, *NbPOD3* and *NbXTH*), transcriptional regulation (*NbNAC90*), glycosylation‐associated metabolism (*NbUGT7*) and stress‐associated responses (*NbSN1*). At 10 dpi, silencing of *NbNDPK2*, *NbERF5*, *NbPOD3*, *NbNAC90*, *NbPAL1*, *NbChiB* and *NbRbohD* resulted in more severe disease symptoms than those observed in control plants, whereas *NbXTH*‐ and *NbUGT7*‐silenced plants showed only mild symptom enhancement and *NbSN1*‐silenced plants showed no obvious difference from the control (Figure 6D, Figure S12). RT‐qPCR analysis confirmed efficient silencing of the target genes, with knockdown efficiencies ranging from 69% to 97% (Figure 6E). Consistent with the symptom phenotypes, TYLCV DNA accumulation and CP levels were increased to different extents in several silenced lines, as determined by RT‐qPCR and western blot analyses, respectively (Figure 6F,G). Collectively, these VIGS results indicate that the candidate genes identified from the *NtSGRL1*‐responsive transcriptome are not merely transcriptional markers but include multiple functional contributors to antiviral defence. The differential effects among the silenced genes further suggest that *NtSGRL1*‐mediated resistance is unlikely to depend on a single downstream effector; rather, it involves coordinated activation of several defence‐related modules, particularly ROS/redox regulation, hormone‐associated defence signalling, phenylpropanoid metabolism and cell wall‐associated responses.

Notably, several defence‐associated genes that showed reduced expression following viral infection in WT plants displayed higher basal expression in the *Ntsgrl1* mutant. Together, these results indicate that loss of *NtSGRL1* is associated with a distinct basal defence‐related transcriptional state involving genes annotated to MAPK signalling, hormone responses, and phenylpropanoid biosynthesis. These molecular changes are consistent with, and may contribute to, the enhanced antiviral phenotype of *Ntsgrl1* plants.

### 2.7 Loss of NtSGRL1 Confers Broad‐Spectrum Antiviral Resistance

To further evaluate the role of *Ntsgrl1* in antiviral defence, we assessed the response of OE, WT, and *Ntsgrl1* mutant plants to multiple viruses, including tomato spotted wilt virus (TSWV), potato virus Y (PVY) and TMV. At 14 dpi with TSWV infection, OE plants exhibited severe chlorosis, leaf deformation, and stunted growth, whereas *Ntsgrl1* mutant plants showed markedly attenuated symptoms compared with WT (Figure 7A). Consistently, quantification of viral RNA revealed significantly higher TSWV accumulation in OE plants, while both *Ntsgrl1* mutant lines (#1 and #14) displayed a dramatic reduction in viral levels relative to WT (Figure 7B). Western blot analysis further confirmed reduced accumulation of viral CP in the mutant plants (Figure 7C). At 14 dpi with PVY, vein necrosis was evident in both OE and WT plants, but absent in the *Ntsgrl1* mutant. Moreover, OE plants developed more severe vein necrosis than WT, suggesting enhanced susceptibility (Figure 7A). Viral accumulation analysis demonstrated a substantial decrease in PVY levels in the mutant lines, accompanied by reduced CP accumulation (Figure 7D,E). Consistent results were also observed for TMV infection. OE plants displayed increased susceptibility with severe mosaic symptoms, whereas *Ntsgrl1* mutant plants showed attenuated symptoms and significantly lower viral accumulation compared with WT (Figure S13).

**FIGURE 7:**
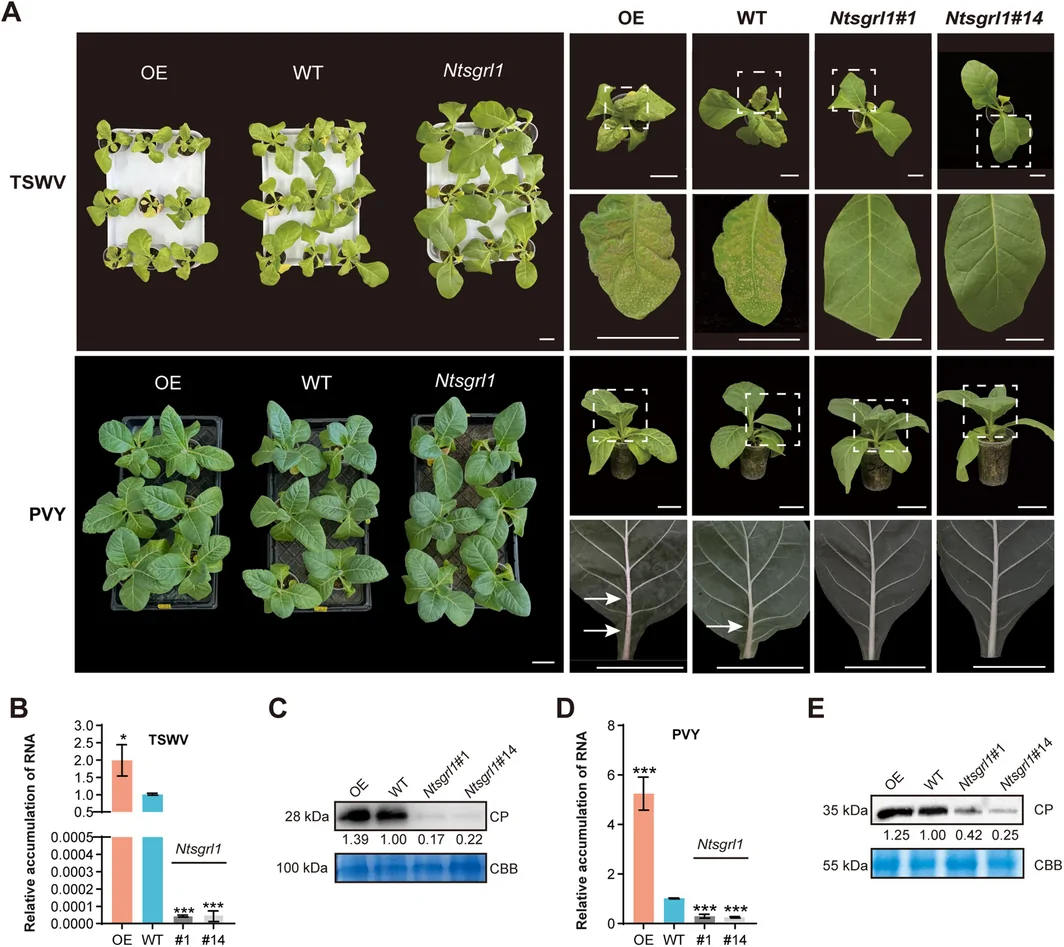
Knockout of *NtSGRL1* enhances tobacco resistance to TSWV and PVY. (A) Disease symptoms of overexpression (OE), wild type (WT), and *Ntsgrl1* mutant plants following TSWV and PVY infection. Representative images show whole plants and magnified views of symptomatic leaves. Scale bar = 5 cm. (B, C) Viral accumulation in tobacco leaves at 14 days post‐inoculation (dpi) with TSWV was determined by western blot and reverse transcription‐quantitative PCR (RT‐qPCR) analyses (*n* = 3). (D, E) Viral accumulation in tobacco leaves at 14 dpi with PVY was determined by western blot and RT‐qPCR analyses (*n* = 3). Scale bar = 5 cm. CP, coat protein; CBB, Coomassie Brilliant Blue. Results are presented as the mean ± SD. Asterisks indicate statistical difference between treatments, determined by the two‐tailed *t*‐test (*p < 0.05, ***p < 0.001).

Taken together, these results demonstrate that loss of *Ntsgrl1* confers enhanced resistance to multiple viruses, whereas its overexpression promotes viral susceptibility, indicating that *Ntsgrl1* functions as a negative regulator of antiviral resistance.

## 3 Discussion

Chloroplasts serve not only as photosynthetic organelles but also as important regulators of plant immunity. In this study, we identified NtSGRL1, a member of the STAY‐GREEN family, as a negative regulator of antiviral resistance in tobacco. Although SGR proteins are primarily characterized as regulators of Chl degradation, leaf senescence, and photosystem remodelling (Wang et al. 2019; Jiang and Zhou 2023), increasing evidence indicates that SGR/SGRL proteins have broader roles in plant development and stress responses (Sakuraba et al. 2014; Ouyang et al. 2023; Uluisik et al. 2022; Li et al. 2024). Consistent with this expanded functional spectrum, transient overexpression of *NtSGRL1* promoted chlorosis and reduced Chl accumulation, whereas knockout of *NtSGRL1* increased Chl content and photosynthetic performance. Loss of *NtSGRL1* also enhanced early seedling growth, indicating that NtSGRL1 influences chloroplast‐associated physiology beyond its established role in Chl catabolism. The limited response of *NtSGRL1* to ABA and MeJA compared with *NtSGR1a* further suggests functional diversification among tobacco SGR family members (Tanaka and Ito 2025).

The developmental effects caused by *NtSGRL1* disruption were stage dependent. Enhanced growth was mainly observed during early seedling development, whereas differences in mature plant architecture and thousand‐seed weight were relatively limited. Unlike classical stay‐green mutants, *Ntsgrl1* plants did not display abnormal persistence of green leaves or obvious developmental defects (Sakuraba et al. 2015; Shimoda et al. 2016; Xie et al. 2025). These results suggest that NtSGRL1 contributes to early chloroplast establishment and photosynthetic regulation rather than acting as a major determinant of whole‐plant development. Importantly, enhanced antiviral resistance in *Ntsgrl1* plants was not accompanied by an apparent growth penalty under the conditions tested, suggesting that *NtSGRL1* disruption may represent a strategy to improve immunity while maintaining basic plant performance.

A major finding of this study is that NtSGRL1 functions as a susceptibility factor for multiple viral pathogens. Knockout of *NtSGRL1* reduced accumulation of TYLCV, TSWV, PVY and TMV, whereas overexpression increased susceptibility to TYLCV. Because these viruses differ substantially in genome organization and infection strategies, the broad‐spectrum resistance phenotype suggests that *NtSGRL1* likely regulates host processes shared among different viral infections rather than virus‐specific mechanisms (Boualem et al. 2016; Rajwade et al. 2020; Wu et al. 2024). However, most mechanistic analyses in this study were performed using TYLCV infection, and whether identical downstream pathways contribute to resistance against other viruses remains to be investigated.

The antiviral function of NtSGRL1 may be associated with its role in chloroplast‐associated signalling. Chloroplasts participate in immune regulation through ROS production, hormone metabolism and retrograde signalling. The observation that NtSGRL1 localizes to both chloroplasts and nuclei raises the possibility that NtSGRL1 may participate in communication between these compartments. Consistent with this possibility, increasing evidence indicates that several chloroplast‐associated proteins can function as dual‐localized regulators involved in retrograde signalling between chloroplasts and the nucleus (Singh et al. 2015). Although NtSGRL1 exhibited transcriptional activation activity in yeast, current evidence is insufficient to define it as a transcription factor or demonstrate direct transcriptional regulation in planta. Therefore, NtSGRL1 should be considered a chloroplast‐associated protein with potential nuclear regulatory activity rather than a confirmed nuclear transcriptional regulator. Identification of NtSGRL1‐interacting proteins and determination of the functional significance of its dual localization will be required to clarify this possibility.

The relationship between NtSGRL1‐mediated immunity and plant growth provides additional insight into the regulation of the growth–defence balance. Plants must optimize resource allocation among growth, reproduction and defence, and excessive immune activation often results in reduced biomass accumulation or yield penalties due to increased metabolic costs (Li et al. 2026; Ding et al. 2024; Derbyshire et al. 2024; Sofi et al. 2026). Interestingly, despite enhanced antiviral resistance, *Ntsgrl1* plants maintained normal development and showed increased Chl accumulation, improved photosynthetic capacity, and enhanced early seedling vigour. These findings suggest that *NtSGRL1* disruption may enhance immune preparedness without triggering a substantial growth trade‐off. One possibility is that altered chloroplast activity in *Ntsgrl1* plants simultaneously supports photosynthetic efficiency and defence‐associated signalling, thereby allowing enhanced resistance while preserving growth capacity. Nevertheless, whether this favourable growth‐defence balance persists under field conditions, prolonged pathogen exposure, or diverse environmental stresses remains to be determined.

The relatively modest changes in Chl‐related traits compared with the pronounced antiviral phenotype further suggest that the biological function of *NtSGRL1* extends beyond pigment metabolism. Although *NtSGRL1* disruption alters chloroplast physiology, the enhanced resistance phenotype is likely mediated through broader defence‐associated reprogramming rather than through changes in Chl accumulation alone. Consistent with this interpretation, transcriptomic analysis revealed extensive transcriptional reprogramming in *Ntsgrl1* plants during TYLCV infection. Differentially expressed genes were significantly enriched in pathways associated with defence responses, oxidative regulation, MAPK signalling, phenylpropanoid metabolism, and cell wall‐associated processes. Functional analyses of selected candidate genes further demonstrated that these transcriptional changes contribute to antiviral resistance, indicating that *NtSGRL1* does not regulate immunity through a single downstream effector but rather influences a broader defence regulatory network.

ROS‐associated signalling represented one of the major immune signatures associated with *NtSGRL1* disruption. Increased expression of *RbohD* and *NDPK2*, together with elevated H_2_O_2_ accumulation and MAPK phosphorylation, indicates that loss of *NtSGRL1* activates a ROS–MAPK signalling module involved in defence amplification. *RbohD*‐mediated ROS production and *NDPK2*‐dependent redox signalling have been shown to promote downstream immune activation, including calcium signalling, MAPK activation, and stress adaptation (Moon et al. 2003; Meng and Zhang 2013; Lukan et al. 2020; Zhang 2020). These results suggest that enhanced ROS signalling may represent an important component underlying the increased antiviral resistance of *Ntsgrl1* plants. However, whether ROS activation acts as a primary driver of resistance or is partially coupled with altered chloroplast‐associated metabolism requires further genetic validation.

Hormonal reprogramming further supports the involvement of multiple defence regulatory layers following *NtSGRL1* disruption. Changes in ABA‐, JA‐ and ET‐associated genes, together with altered ABA, ACC and JA contents, indicate that *NtSGRL1* affects the balance of stress‐related hormone networks. Notably, persistent activation of *ERF1* and *ChiB*, accompanied by reduced *MYC2*‐associated JA signalling after infection, suggests a preferential shift toward an *ERF*/ethylene‐associated defence programme rather than the *MYC2*‐dominated JA branch. Because *ERF1* integrates JA and ET signalling to regulate defence gene expression, this hormonal reconfiguration may enhance immune activation while altering the balance between defence and growth‐associated responses (Lorenzo et al. 2003; van Loon et al. 2006). Together with elevated ROS–MAPK activity, this hormone shift provides a potential regulatory framework explaining the enhanced antiviral phenotype of *Ntsgrl1* mutants.

In addition to signalling changes, transcriptional activation of phenylpropanoid metabolism and cell wall‐associated pathways suggests a possible reinforcement of structural and chemical barriers during infection. Phenylpropanoid‐derived compounds contribute to pathogen resistance through antimicrobial activity and cell wall modification, while cell wall dynamics influence virus movement between adjacent cells (Wang 2021; German et al. 2023). Although further biochemical and cellular analyses are required to determine their direct contribution to viral restriction, these pathways likely represent additional components of the enhanced defence state triggered by *NtSGRL1* disruption.

From a mechanistic perspective, our findings support a model in which *NtSGRL1* negatively regulates antiviral immunity through coordination between chloroplast‐associated physiology and defence‐related transcriptional networks. Loss of *NtSGRL1* alters Chl metabolism, ROS‐associated responses, MAPK activation, hormone‐related pathways and defence gene expression, collectively contributing to enhanced antiviral resistance. However, the current study does not determine whether *NtSGRL1* directly controls immune genes, whether chloroplast‐derived signals are required for resistance, or which downstream pathway is essential for the phenotype. Future studies integrating protein interaction analyses, genetic epistasis approaches, and chloroplast‐to‐nucleus signalling assays will be important for resolving the molecular mechanism.

Another notable feature of the *Ntsgrl1* transcriptome was the higher basal abundance of several defence‐associated transcripts. Genes (e.g., *XTH*) that are typically induced upon infection in WT plants already showed elevated basal expression in the mutant. This apparent discrepancy reflects a key regulatory principle: defence capacity depends not only on fold‐change responsiveness but also on basal transcriptional readiness. Upon infection, feedback regulation may attenuate further induction to prevent excessive immune activation and metabolic cost (Hönig et al. 2023; Giovanini et al. 2026). Thus, *NtSGRL1* likely functions upstream of this priming state by repressing basal activation of defence‐related networks.

From an applied perspective, *NtSGRL1* represents a potential susceptibility gene for engineering virus resistance in tobacco. Disruption of *NtSGRL1* enhanced resistance to multiple viruses without obvious negative effects on major growth traits or thousand‐seed weight under controlled conditions. This feature is particularly attractive because many resistance‐enhancing mutations are associated with growth–defence trade‐offs (Gao et al. 2024). Nevertheless, evaluation under field conditions, diverse genetic backgrounds, prolonged pathogen pressure, and reproductive performance will be required before its breeding potential can be fully assessed.

In summary, this study expands the functional understanding of SGR family proteins by demonstrating that NtSGRL1 connects chloroplast‐associated processes with antiviral immunity. Our findings identify *NtSGRL1* as a negative regulator of broad‐spectrum viral resistance and reveal a previously under‐appreciated role of chloroplast‐related regulation in shaping plant immune capacity. These results provide new insights into the relationship between chloroplast function, immune priming, and growth–defence balance, while highlighting NtSGRL1 as a promising target for future antiviral resistance improvement.

## 4 Experimental Procedures

### 4.1 Plant Materials and Growth Conditions

*Nicotiana benthamiana* lab strain and *N. tabacum* flue‐cured cultivars K326 were cultivated in controlled growth chambers maintained at 25°C ± 2°C, with a 16‐h light/8‐h dark photoperiod, 60% relative humidity, and a photosynthetic photon flux density (PPFD) of c. 150 μmol m^−2^ s^−1^.

Stable knock‐out and OE lines were generated by gene editing and overexpression approaches, respectively. CRISPR/Cas9 gene editing was carried out using the previous methods (Li et al. 2013). For the full‐length coding sequence (CDS) of the *NtSGRL1* gene, two 19 nt optimal target sites that specifically target the tobacco endogenous gene *NtSGRL1* were screened using the online target design tool (http://crispor.tefor.net/). These target sites were ligated into the general gene‐editing vector pHSE401 to construct the targeting vector pHSE‐*Ntsgrl1*. To construct *NtSGRL1*‐overexpressing transgenic tobacco plants, the full‐length CDS of *NtSGRL1* was cloned from the tobacco genome and then inserted into the pSuper1300‐GFP vector. The plasmid was transformed into tobacco by *Agrobacterium tumefaciens* LBA4404. The *Ntsgrl1* mutants were produced by selecting homozygous progeny by PCR analysis. Transgenic plants were screened and regenerated on MS medium supplemented with 20 mg/L hygromycin (Hyg). Resistant plantlets were further propagated and transferred to soil for subsequent analysis. For CRISPR/Cas9‐edited lines, homozygous mutants were identified by PCR‐based genotyping. Representative mutant lines, including *Ntsgrl1*#1, *Ntsgrl1*#8 and *Ntsgrl1*#14, were selected for further analysis. These lines carried distinct insertion or deletion mutations at the target site.

### 4.2 Mechanical Inoculation and Agroinoculation

The infectious clone pCambia2300‐TYLCV‐BJ (GenBank accession no. MN432609) was kindly provided by Prof. Xueping Zhou (Institute of Plant Protection, Chinese Academy of Agricultural Sciences). TMV, PVY and TSWV were maintained in our laboratory. TYLCV represents a DNA virus, whereas TMV, PVY, and TSWV are RNA viruses, enabling comparative analysis of NtSGRL1‐mediated responses to different viral types and evaluation of its potential role in broad‐spectrum antiviral resistance.

For mechanical transmission, 1 g of young leaf tissues from the infected plants was ground in 10 mL of 0.1 M phosphate buf (pH 7.2) containing 0.5% celite to prepare virus inoculum. Crude leaf extracts were mechanically inoculated onto different tobacco varieties and genome‐edited lines. Experiments were repeated three or four times, each with sample sizes ranging from 5 to 24 plants (biological replicates) per group to ensure statistical robustness.

*Agrobacterium tumefaciens* cultures harbouring the TYLCV infectious clone were incubated overnight at 28°C, harvested the following morning, and resuspended in infiltration buffer containing 10 mM MES (pH 5.6), 10 mM MgCl_2_, and 150 μM acetosyringone to an OD_600_ of 1.0. After incubation at room temperature for 3 h, the suspension was infiltrated into the third leaf of tobacco plants using a 1 mL sterile needleless syringe.

### 4.3 Identification and Bioinformatics Analysis of NtSGR Family Members

Based on the *N. tabacum* K326 genome data, we first conducted a systematic identification of NtSGR family members. Using the reported SGR protein sequences from *Arabidopsis* as a reference, we BLAST searched the *N. tabacum* genome (https://solgenomics.net/organism/Nicotiana_tabacum/genome) in the NCBI database and conducted a preliminary screening of candidate sequences based on sequence similarity, conserved domain information, and protein integrity (Li et al. 2024; Ouyang et al. 2023; Uluisik et al. 2022). After removing redundant and obviously incomplete sequences, the NtSGR family members were named based on their phylogenetic correspondence with homologous SGR proteins in *Arabidopsis*.

We used the ExPASY server (http://web.expasy.org/protparam/) to compute the sequence length, theoretical isoelectric point (pI), grand average of hydropathicity (GRAVY) and molecular weight (MW) of the NtSGR proteins (Table S1).

The multiple sequence alignments for SGR proteins from *N. tabacum* , *A. thaliana* , and other crops were performed by using ClustalX software with default parameters (Larkin et al. 2007). MEGA 11.0 software was used to construct a phylogenetic tree by using the neighbour‐joining (NJ) method with 1000 bootstrap replicates. Gene structures (exon‐intron organization) were analysed using GSDS v. 2.0 (http://gsds.cbi.pku.edu.cn). Conserved motifs were identified using MEME with a maximum of 10 motifs (Bailey et al. 2009).

Multiple sequence alignments of NtSGR and AtSGR proteins were performed and visualized using ESPript v3.2 (https://espript.ibcp.fr/ESPript/cgi‐bin/ESPript.cgi). The amino acid sequences of NtSGRL1, NtSGR1a and NtSGR1b were submitted to the SWISS‐MODEL server (https://swissmodel.expasy.org/) for tertiary structure prediction, and structural differences were compared among the three proteins.

### 4.4 Subcellular Localization of NtSGRs

The CDS of the *NtSGRL1*, *NtSGR1a* and *NtSGR2a* lacking stop codons were ligated into the 35S::GFP vector to construct the NtSGRL1‐GFP, NtSGR1a‐GFP, and NtSGR2a‐GFP fusion proteins. The 35S::GFP (an empty vector) was used as a negative control. The subcellular localization analysis was performed by introducing the constructed vector into young tobacco leaves by *A. tumefaciens* ‐mediated transformation (Meng et al. 2018). The GFP protein signals, RFP nuclear marker signals and chloroplast autofluorescence signals were detected under a laser scanning confocal microscope (Olympus) as described previously (Yoo et al. 2007; Wang et al. 2023).

### 4.5 Functional Analysis of NtSGRL1

The vectors from subcellular localization analysis were reused to perform the transient overexpression analysis (Yang et al. 2019). The experiment was performed in 6‐week‐old tobacco plants by selecting healthy leaves at the fourth internode (counting from the shoot tip) for bacterial culture inoculation. Chl contents, Fv/Fm, and SPAD value in transformed tobacco leaves were determined according to previously described methods (Tan et al. 2019). RT‐qPCR was employed to monitor the expression dynamics of *NtSGRL1*, *NtSGR1a* and *NtSGR2a* during natural senescence, while simultaneously detecting changes in the expression of these genes under exogenous ABA and MeJA treatment conditions.

### 4.6 Transcriptional Activation Assay in Yeast

The transcriptional activation activity of *NtSGRL1* was evaluated using a yeast‐based transcriptional activation assay. The full‐length CDS of *NtSGRL1* was fused to the GAL4 DNA‐binding domain (BD) in the pGBKT7 vector and transformed into the AH109 yeast strain. The empty pGBKT7 vector and pGBKT7‐VP16 were used as the negative and positive controls, respectively, and a pGBKT7‐VP16‐NtSGRL1 construct was included to assess whether fusion with the VP16 activation domain affected NtSGRL activity. Transformants were first selected on SD/−Trp medium and subsequently spotted onto SD/−Trp−His/X‐α‐Gal and SD/−Trp−His−Ade/X‐α‐Gal media. Plates were incubated at 30°C for 3–5 days, and transcriptional activation activity was evaluated based on yeast growth and X‐α‐Gal colour development.

### 4.7 Evaluation of Agronomic Traits and Photosynthetic Traits in the Field

The *Ntsgrl1*, *NtSGRL1*‐OE and WT plants were grown in the experimental fields at the back hill of Shenyang Agriculture University. Each cultivar was planted in 2 rows with a row length of 30 m and the width and length between two adjacent plants were 1 and 0.5 m. Two rows of WT were planted on each side as border rows. Ten plants were randomly selected from each row to determine agronomic and photosynthetic traits. During the seedling stage, the growth vigour and leaf expansion of plants of different genotypes were evaluated. During the field growth stage, agronomic traits—including leaf senescence rate (LSR), plant height, stem circumference, fresh weight, and biomass—were measured in vigorous and mature plants.

A portable photosynthetic measurement system (LI‐6400) was used to determine photosynthetic parameters such as net photosynthetic rate, stomatal conductance, and transpiration rate. The SPAD value was measured with the middle portion of the leaf using a handheld SPAD Chl meter (Minolta SPAD‐502 plus). Chl was extracted from the leaves with extraction solution (acetone:ethanol:water = 4.5:4.5:1, v/v/v), and then Chl content per gram fresh weight (FW) of the leaf was determined as described previously (Xie et al. 2022).

### 4.8 RNA‐seq and Data Analysis

The upper leaf collected from at least five *N. tabacum* plants were pooled for RNA‐seq with three biological replicates in WT, WT_V, *Ntsgrl1* and *Ntsgrl1*_V plants. Three biological replicates were performed for each sample. Total RNA was extracted from the pooled samples using a Total RNA Extraction Kit (Tiangen). RNA sequencing was performed using the Illumina Novaseq X Plus platform at the Beijing Novogene Technology Co. Ltd., in Beijing, China. For data analysis, clean reads were aligned to the reference genome K326 Release 1.0 using HISAT2 v.2.0.5 (Mortazavi et al. 2008). FeatureCounts v.1.5.0‐p3 was used for quantitative analysis of genes. The relative gene expression levels were normalized as fragments per kilobase of transcript per million mapped reads (FPKM). DESeq2 was used for differential expression analysis, and false discovery rate (FDR)‐adjusted *p* < 0.05, |log_2_ fold change| ≥ 1 were used as the criteria to screen for DEGs. GO functional enrichment analysis and KEGG pathway enrichment analysis of DEGs were performed using the clusterProfiler package. KEGG pathway enrichment analyses of DEGs were performed by clusterProfiler v.3.8.1. The original RNA‐seq data were deposited to the Sequence Read Archive of the NCBI with Bioproject ID: PRJNA1466156.

### 4.9 Virus‐Induced Gene Silencing (VIGS) Assays

The TRV‐VIGS assays were performed according to a previous report (Guo et al. 2022). In this study, 10 genes were silenced using a tobacco rattle virus (TRV)‐based VIGS vector in *N. benthamiana*. The fragments were cloned from *N. benthamiana* cDNA using specific primers with homologous arms (Table S11). The upper two non‐infiltrated *N. benthamiana* leaves were mechanically inoculated with TYLCV. The first systemically infected leaves were collected at 7 dpi.

### 4.10 RT‐qPCR

Total RNA was extracted from the first systemically infected tobacco leaves by TRIzol Reagent (Vazyme), which was reversely transcribed to cDNA by HiScript III RT SuperMix (+gDNA wiper) (Vazyme). Real‐time qPCR was performed using ChamQ SYBR qPCR Master Mix (Vazyme) with a StepOne Plus real‐time PCR system (Applied Biosystems). The relative gene expression was calculated by the 2^−ΔΔCt^ method (Livak and Schmittgen 2001). *N. benthamiana Actin* gene (AY179605.1) *and N. tabacum Actin* gene (AB158612.1) were used as internal controls. The specific primers for qPCR detection were designed according to gene sequences of *N. benthamiana* and *N. tabacum* (Table S12). The experiments were performed with three biological replicates.

### 4.11 Western Blot Analysis

Total proteins were extracted from leaf samples using a Plant Protein Extraction Kit (Coolaber), whereas proteins used for phosphorylated MAPK detection were extracted with EDTA‐free high‐efficiency RIPA tissue/cell lysis buffer supplemented with PMSF (Coolaber). Equal amounts of protein samples were separated by 12% SDS‐PAGE and transferred onto 0.22‐μm polyvinylidene fluoride (PVDF) membranes (Sangon Biotech).

For viral protein detection, membranes were immunoblotted with monoclonal antibodies against TYLCV, TSWV, PVY, or TMV, followed by incubation with a horseradish peroxidase (HRP)‐conjugated anti‐mouse IgG secondary antibody (Solarbio). Phosphorylated MAPKs were detected using a Phospho‐p44/42 MAPK (Erk1/2) (Thr202/Tyr204) rabbit monoclonal antibody (#4377; Cell Signalling Technology), followed by an HRP‐conjugated anti‐rabbit IgG secondary antibody (Lee et al. 2018). The large subunit of ribulose‐1,5‐bisphosphate carboxylase/oxygenase (RuBisCO), visualized by Coomassie Brilliant Blue staining of 12% SDS–PAGE gels, was used as the loading control.

Protein signals were developed using an enhanced chemiluminescence (ECL) substrate (Millipore) and detected using a chemiluminescence imaging system (Tanon). Band intensities were quantified using image‐analysis software.

### 4.12 Detection of ROS

H_2_O_2_ accumulation in tobacco leaves was detected using DAB staining (Coolaber). Leaves were vacuum‐infiltrated with DAB staining solution (−0.1 MPa, 30 min), incubated in the dark at room temperature until brown precipitates developed, and subsequently decolorized in 95% ethanol at 70°C–80°C until chlorophyll was completely removed. After rehydration in distilled water, stained leaves were photographed, and DAB staining intensity was quantified using ImageJ.

Total ROS levels were determined using a DCFH‐DA‐based assay kit (Coolaber). Briefly, 0.1 g of frozen leaf tissue was homogenized in 1 mL of ice‐cold extraction buffer and centrifuged at 12,000 *g* for 10 min at 4°C. The supernatant (200 μL) was mixed with 2 μL DCFH‐DA probe in a black 96‐well plate and incubated at 37°C for 30 min in the dark. Fluorescence was measured using a microplate reader (Ex 488 nm/Em 525 nm). Relative ROS level was calculated as  ΔF/(W × V₁/V), where W represents the fresh weight of the tissue sample (g), V₁ is the volume of supernatant used in the reaction (μL), and V is the total volume of extraction buffer (μL).

### 4.13 Data Processing and Statistical Analysis

All the data were analysed using SPSS Statistics 25 software (IBM Inc.). The differences among groups were analysed through a two‐tailed *t*‐test and one‐way analysis of variance (Duncan).

## Author Contributions

**Ming Gu:** conceptualization, data curation, writing – original draft, visualization, formal analysis, methodology, investigation, software. **Kexin Cao:** validation, formal analysis, investigation. **Jiamin Yu:** project administration, resources, investigation. **Dongyang Liu:** investigation, project administration, resources. **Bin Zhao:** data curation, formal analysis. **Siyu Sun:** data curation, formal analysis. **Zhenhong Zhu:** data curation, formal analysis. **Mengnan An:** writing – review and editing, validation. **Zihao Xia:** writing – review and editing, project administration, funding acquisition, resources. **Zhiping Wang:** project administration, writing – review and editing, funding acquisition, validation, supervision, methodology. **Yuanhua Wu:** methodology, project administration, writing – review and editing, funding acquisition, resources, supervision.

## Funding

This research was supported by Mechanism and Innovation & Application of Green Prevention and Control Technology of vector‐transmitted Plant Viral Diseases (SCYC202605).

## Conflicts of Interest

The authors declare no conflicts of interest.

## Supporting information


**Figure S1:** Multiple sequence alignment results of tobacco and *Arabidopsis* SGR proteins.

**Figure S2:** Predicted three‐dimensional structures of representative NtSGR proteins.

**Figure S3:** Transient overexpression analysis of NtSGR homologues in tobacco leaves. (A) Relative expression levels of *NtSGR* homologues in transient overexpression assays. (B) Phenotypic changes of tobacco leaves transiently expressing empty vector (EV), *NtSGR1a*, *NtSGR2a* or *NtSGRL1*. Representative images were taken at 60 h after infiltration.

**Figure S4:** Expression patterns of *NtSGR1a*, *NtSGR2a* and *NtSGRL1* in response to (A) abscisic acid (ABA) and (B) methyl jasmonate (MeJA) treatments.

**Figure S5:** CRISPR/Cas9‐mediated mutagenesis of *NtSGR1a*, *NtSGR2a* and *NtSGRL1* in tobacco. (A) Schematic representation of the *NtSGR1a* genomic structure and CRISPR/Cas9 target site. (B) Gene structure of *NtSGR2a* and CRISPR/Cas9‐induced mutation. (C) Gene structure of *NtSGRL1* and multiple independent CRISPR alleles (*Ntsgrl1*#14). Exons are shown as green boxes and introns as black lines. Target sites are indicated by red triangles. Wild‐type amino acid sequences and mutant‐derived truncated peptides are shown below each gene structure.

**Figure S6:** Analysis of mutation types and growth phenotypes in *NtSGRL1* knockout lines. (A) Schematic illustration of the sgRNA target site and protein sequence of wild type (WT), *NtSGRL1* and the allele from CRISPR/Cas9 T_2_ mutant lines (*Ntsgrl1*#1, 8, 14). Dash presents the deletion sequences, the blue arrow indicates the sgRNA target site and the asterisk presents the termination of the protein sequence. (B) Schematic of the NtSGRL1‐GFP overexpression construct. (C) Phenotypic comparison of WT, OE#1 and *Ntsgrl1* #14 plants at the seedling and vegetative stages. Upper panels show seedlings grown on plates; lower panels show plants grown in soil. Total leaf area is indicated below each genotype. Scale bar = 5 cm.

**Figure S7:** Agronomic traits of wild type (WT) and *Ntsgrl1* plants under field conditions. (A) Plant height. (B) Stem circumference. (C) Internode length. (D) Total leaf number. (E) Maximum leaf area. Individual data points are shown as dots, and bars represent mean ± SD (*n* = 20). Statistical significance among genotypes and growth stages was determined using one‐way ANOVA followed by Tukey's multiple comparison test.

**Figure S8:** Photosynthetic, biomass, and yield‐related traits of wild type (WT) and *Ntsgrl1* plants. (A) Chlorophyll content. (B) Stomatal conductance (Gs). (C) transpiration rate (E). (D) Leaf biomass. (E) Leaf fresh weight. (F) Thousand‐seed weight. Individual data points are shown as dots, and bars represent mean ± SD (*n* = 20). Statistical significance among genotypes and growth stages was determined using one‐way ANOVA followed by Tukey's multiple comparison test.

**Figure S9:** Quality assessment and reproducibility analysis of RNA‐seq datasets. (A) Quality assessment and reproducibility analysis of RNA‐seq datasets. (B) Hierarchical clustering heatmap based on Pearson correlation coefficients among all samples, indicating high reproducibility within replicates and clear separation between groups.

**Figure S10:** Functional enrichment analysis of differentially expressed genes between wild type (WT) and *Ntsgrl1* mutant. (A) Gene Ontology (GO) enrichment analysis of differentially expressed genes (DEGs) identified between WT and *Ntsgrl1* mutant plants. Enriched GO terms are categorized into Biological Process (BP), Cellular Component (CC) and Molecular Function (MF), and are ranked by −log_10_ (*p* value). (B) KEGG pathway enrichment analysis of DEGs between WT and *Ntsgrl1*. Enriched pathways are displayed as bubble plots, where bubble size indicates the number of enriched genes and colour represents statistical significance (*p* value).

**Figure S11:** Reactive oxygen species (ROS) accumulation, MAPK phosphorylation and defence‐related hormone levels in wild type (WT) and *Ntsgrl1* plants following TYLCV infection. (A) Representative 3,3′‐diaminobenzidine (DAB) staining of leaves from mock‐treated and TYLCV‐infected WT and Ntsgrl1 plants. The percentage of DAB‐stained leaf area is indicated below each image. (B) ROS generation rate in WT and *Ntsgrl1* plants with or without TYLCV infection. (C) Immunoblot analysis of phosphorylated MAPKs (p‐P44/42 MAPK). Coomassie brilliant blue (CBB) staining was used as a loading control. (D–F) Contents of abscisic acid (ABA) (D), jasmonic acid (JA) (E) and the ethylene precursor 1‐aminocyclopropane‐1‐carboxylic acid (ACC) (F) in WT and *Ntsgrl1* plants with or without TYLCV infection. WT_V and *Ntsgrl1*_V indicate TYLCV‐infected WT and *Ntsgrl1* plants, respectively. Data are presented as the mean ± SD (*n* = 3), with statistical significance indicated as *p < 0.05, **p < 0.01, ***p < 0.001.

**Figure S12:** Phenotypes of tobacco plants subjected to virus‐induced gene silencing (VIGS) of candidate genes at 10 days post‐inoculation. Plants infiltrated with TRV‐*NbPDS* served as the positive control. TRV‐*NbGFP* was used as the negative control.

**Figure S13:** *Ntsgrl1* modulates resistance to TMV infection. (A) Disease symptoms of overexpression (OE), wild type (WT), and *Ntsgrl1* mutant plants following TMV infection. (B) Quantification of TMV accumulation by reverse transcription‐quantitative PCR in OE, WT and *Ntsgrl1* mutant plants. Data are shown as means ± SD (*n* = 3), with statistical significance indicated as **p < 0.01, ***p < 0.001. (C) Western blot analysis of TMV coat protein (CP) accumulation. Coomassie Brilliant Blue staining was used as a loading control.

**Table S1:** Basic characteristics of the SGR genes family in *Nicotiana tabacum* .

**Table S2:** Protein accession numbers of SGR from various crops.

**Table S3:** Annotation of conserved motifs identified in six NtSGR proteins.

**Table S4:** Summary of RNA‐sequencing data quality for the 12 tobacco cDNA libraries.

**Table S5:** Summary of RNA‐seq read mapping to the tobacco reference genome.

**Table S6:** GO terms among differentially expressed genes in the wild type (WT) vs. *Ntsgrl1* comparison.

**Table S7:** KEGG pathways enrichment analysis of differentially expressed genes between wild type (WT) and *Ntsgrl1* mutant.

**Table S8:** Abbreviations of the genes and compounds in Figure 6.

**Table S9:** KEGG pathways enriched by *NtSGRL*1‐dependent TYLCV‐induced genes.

**Table S10:** GO terms enriched by *NtSGRL*1‐dependent TYLCV‐induced genes.

**Table S11:** Reverse transcription‐quantitative PCR validation of the expression levels of selected differentially expressed genes identified by RNA‐seq.

**Table S12:** Reverse transcription‐quantitative PCR specific primers of *Nicotiana benthamiana* and *Nicotiana tabacum*.

## Data Availability

The RNA‐seq transcriptomic data have been deposited to the NCBI Sequence Read Archive (SRA) database under the BioProject accession number PRJNA1466156.
